# Association of Increased Remnant Cholesterol and the Risk of Coronary Artery Disease: A Retrospective Study

**DOI:** 10.3389/fcvm.2021.740596

**Published:** 2021-10-29

**Authors:** Wang Kexin, Ding Yaodong, Gao Wen, Wang Rui, Yang Jiaxin, Liu Xiaoli, Shen Hua, Ge Hailong

**Affiliations:** ^1^Department of Cardiology, Beijing Anzhen Hospital, Capital Medical University, Beijing, China; ^2^Department of Cardiology, Bayannaoer City Hospital, Bayannaoer, China

**Keywords:** coronary artery disease, remnant cholesterol, non-HDL-C, propensity score matching, clinical retrospective study, risk factors

## Abstract

**Background and Aims:** Low-density lipoprotein cholesterol (LDL-C) is the primary target of lipid-lowering therapy in coronary artery disease (CAD). But some patients with the normal levels of LDL-C still suffer from CAD progression and malignant outcomes (e.g., major adverse cardiovascular events [MACEs]), and the mechanism is unclear. The previous prospective studies demonstrated that the remnant cholesterol (RC) and non-high-density lipoprotein cholesterol (non-HDL-C) were capable to predict the risk of CAD. This study evaluated the association between RC and non-HDL-C with the risk of CAD.

**Methods:** In our study, 12,563 patients were enrolled. We categorized patients into four concordance/discordance groups according to the median of RC, LDL-C, and non-HDL-C. Then, we performed a propensity score matching (PSM) strategy. The unadjusted and adjusted multivariate logistic regression models were used to evaluate the relationship between the lipid concentrations.

**Results:** In this study, 8,658 (68.9%) patients were male with a median age of 61 (54 and 67) years. The multivariate logistic regression showed the odds ratio (*OR*) of RC was 1.952 (*CI* = 1.276–2.988, *p* = 0.002). The *OR* of the low RC/high LDL-C group was 0.626 (*CI* = 0.504–0.778, *p* < 0.001) and the *OR* of the low RC/high non-HDL-C group was 0.574 (*CI* = 0.462–0.714, *p* < 0.001). The *p*-values for interaction between the RC and hypertension, diabetes were both < 0.001.

**Conclusion:** Our study showed a significant association between the RC and CAD. The level of RC was more capable to reflect the risk of CAD than LDL-C and non-HDL-C. There was an interaction relationship between RC and age, gender, hypertension, diabetes, in CAD. But we did not find whether there was a relationship between the non-HDL-C and CAD.

## Introduction

Over the past years, coronary artery disease (CAD) has emerged as the leading cause of morbidity and mortality worldwide (1, 2). Great progress has been made in the research of its pathophysiology, and it has been demonstrated that the low-density lipoprotein cholesterol (LDL-C) plays an important role in the process of atherosclerosis, which made it the primary target in lipid-lowering therapy (3–5), such as statins or non-statin agent (e.g., ezetimibe and evolocumab) (6, 7). However, there are still a great number of patients, which have received the treatment above, suffering from disease progression and malignant outcomes (e.g., major adverse cardiovascular events [MACEs]).

Meanwhile, remnant cholesterol (RC) has drawn increasing attention from cardiologists. RC, defined as the cholesterol content of triglyceride-rich lipoproteins (TRLs), consists of very low-density lipoproteins and intermediate-density lipoproteins (VLDL and IDL) in fasting state, and chylomicron remnants in the non-fasting state (8–10). Recently, several studies have demonstrated that the elevated level of RC in serum is one of the significant risk factors for atherosclerosis, and it is RC and non-HDL-C, but not LDL-C, which better reflects the outcome of CAD, independently (10–14). Therefore, in this clinical retrospective study, we aimed to test the hypothesis that the elevated RC and non-HDL-C index can be significant and independent risk factors for CAD and to explore whether the level of RC is more capable to predict the risk of CAD than LDL-C and non-HDL-C.

## Methods

### Study Design and Population

This retrospective study enrolled 12,563 patients, which once underwent coronary angiography from January 1, 2019, to January 21, 2020, at Beijing Anzhen Hospital, Capital Medical University, Beijing, China. Our major exclusion criteria included extreme age (patients <18 or>80 years old), valvular heart disease, cardiomyopathy, severe hepatic or renal dysfunction, a history of stroke or myocardial infarction (MI), malignant tumor, leukopenia or thrombocytopenia, any ongoing inflammatory, in-stent restenosis (ISR), chronic total occlusion (CTO), and the patients whose date of LDL-C was not available. The study was approved by the Beijing Anzhen Hospital Ethics Committee of Capital Medical University, and all the patients gave their advance consent to participate in this study.

### Measurements

The patient demographics data, including gender, age, body mass index (BMI), smoking status, and clinical characteristics, such as hypertension, hypercholesterolemia, diabetes mellitus, and laboratory results, were obtained from the original electronic medical records. We took all the serum samples from the patients after an overnight fasting (>8 h) and stored them at −70°C for laboratory analysis. Fasting blood glucose (FBG), glycosylated hemoglobin (HbA1c), lipid profiles level including total cholesterol (TC), triglycerides (TG), high-density lipoprotein cholesterol (HDL-C), and LDL-C were all measured by the standard laboratory techniques using a chemiluminescence method with a Roche Diagnostics Cobas analyzer Cobas 8000, c702 module (Roche Diagnostics, Shanghai, China). Furthermore, non-HDL-C was estimated as total cholesterol minus HDL-C while remnant-C was calculated as total cholesterol minus LDL-C minus HDL-C. All the blood samples were tested in triplicate under the guidance of the instructions from the manufacturer.

### Diagnostic Criteria

CAD was diagnosed according to the guideline of the European Society of Cardiology (ESC) in 2019 (15). Hypertension was defined as systolic blood pressure (SBP) ≥ 140 mmHg, diastolic blood pressure (DBP) ≥ 90 mmHg, or previously diagnosed hypertension (16). Hypercholesterolemia was defined as TC > 5.18 mmol/L (200 mg/dl) or TG > 1.72 mmol/L (150 mg/dl). Diabetes mellitus was defined as a fasting serum glucose ≥ 7.0 mmol/L or a non-fasting glucose ≥ 11.10 mmol/L according to the WHO guidelines on diabetes (17).

### Statistical Analysis

We calculated the medians of the RC, LDL-C, and non-HDL-C index to divide all the patients into two different groups: low (less than the medians) and high (equal to or greater than the medians). Then, we categorized the patients into the four groups according to a low or high RC index and LDL-C, RC and non-HDL-C, non-HDL-C, and LDL-C as follows: low/low, low/high, high/low, and high/high. The categorical variables were presented as the absolute numbers and percentages while analyzed by the χ^2^-test or Fisher's exact test. The continuous variables were presented as the medians and interquartile range (IQR) (Q25 and Q75) while analyzed by the Kruskal–Wallis test because of skewed distribution. The Spearman's ρ test correlation analyses were also used to investigate the correlation between the RC and other CAD risk factors.

Furthermore, a receiver operating characteristic (ROC) curve was used to identify the cut-off point of RC and divided 12,563 patients into the two groups (less than the medians and equal to or greater than the medians). Then, we performed a propensity score matching (PSM) strategy according to the RC group to reduce the influence of observed imbalances in the clinical baseline characteristics, which adopted a multivariable logistic regression model based on: age, gender, hypertension, hypercholesterolemia, smoking status, diabetes mellitus, BMI, DBP, SBP, white blood cell (WBC), red blood cell (RBC), platelets (PLT), hemoglobin (Hb), prothrombin time (PT), activated partial thromboplastin time (APTT), homocysteine (Hcy), creatinine (CR), uric acid (UA), FBG, HbA1C, hyper-sensitive C-reactive protein (hs-CRP), brain natriuretic peptide BNP, and the matching ratio is 1:1. The multivariate logistic regression analyses were used to adjust confounders and calculate odds ratios (*OR*) and 95% *CI*. Finally, the subgroup analyses were carried out to examine the *p*-value for interaction between the RC and other risk factors after PSM.

All the statistical data analyses were conducted by IBM SPSS software version 26.0 (IBM, NY, USA). A two-tailed *p*-value of < 0.05 was considered statistically significant in our analyses.

## Results

### The Baseline Clinical Characteristics (Total Population)

**Figure 1:**
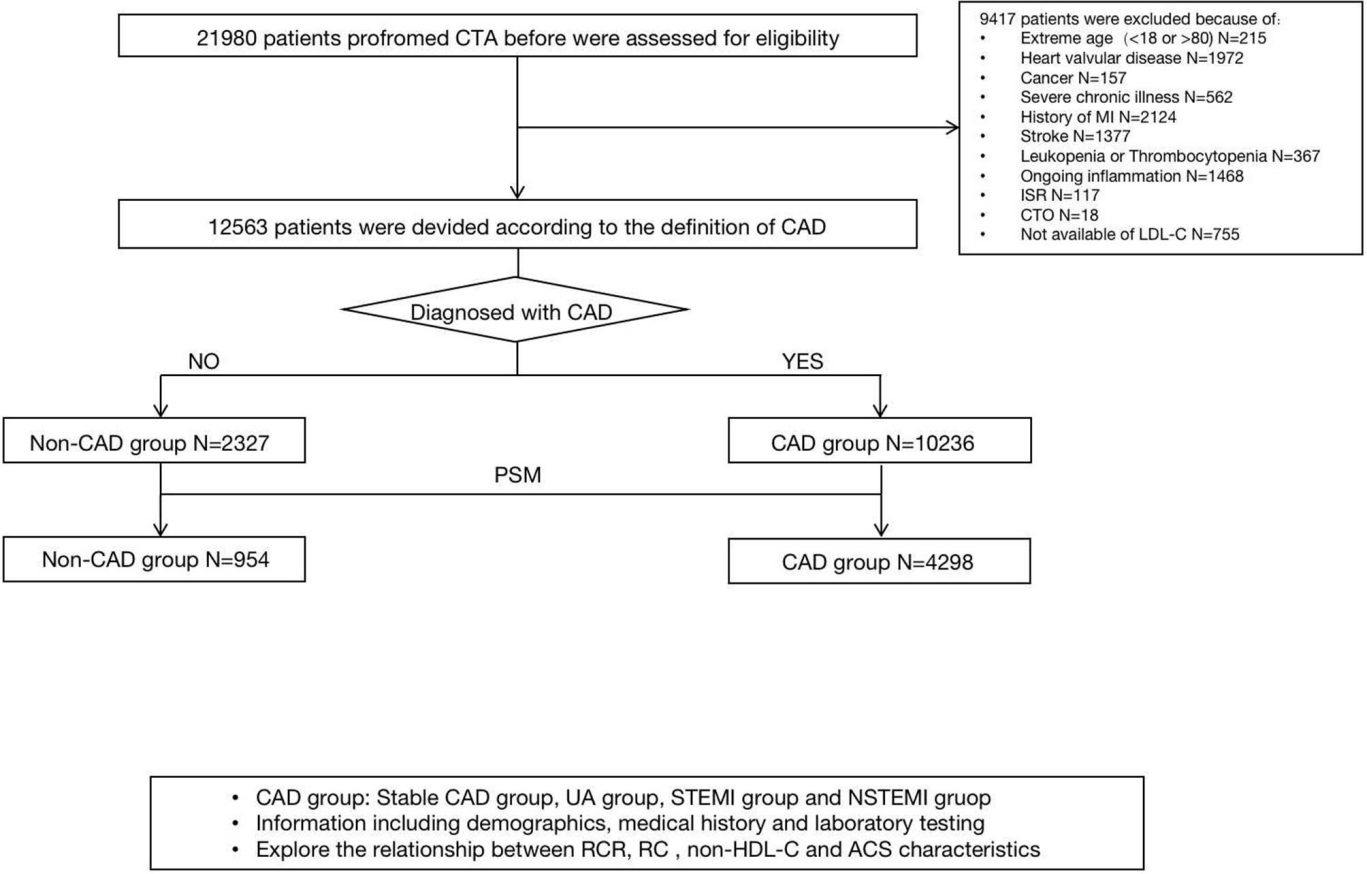
A study flowchart of enrollment.

Among 21,980 patients who underwent coronary angiography before, 12,563 patients were enrolled in the final analyses, with 10,236 in the CAD group and 2,327 in the non-CAD group. The flowchart is shown in Figure 1. The baseline clinical characteristics of all the patients are shown in Table 1. Among the patients enrolled, 8,658 (68.9%) were male with a median age of 61 (54 and 67) years and BMI of 25.86 (23.87, 28.02) kg/m^2^, 62.9% (7,906), and 31.2% (3,920) of whom had hypertension and diabetes. The median values which defined the concordance/discordance groups for RC, LDL-C, and non-HDL-C were 0.51, 2.26, and 2.83 mmol/L, respectively. Moreover, in the clinical presentation, most of the participants were unstable angina (72.1%), while a small part of patients showed non-CAD (16.6%), stable CAD (1.9%), non-ST segment elevation myocardial infarction (5.3%), and ST segment elevation myocardial infarction (4.0%).

**Table 1:** The baseline clinical characteristics and laboratory parameters of the patients according to the remnant cholesterol (RC) and the low-density lipoprotein cholesterol (LDL-C) index categories.

**Variables**	**All Participants** **(*N* = 12,563)**	**RC < Median** **LDL-C < Median group** **(*n* = 3,596)**	**RC < Median** **LDL-C≥Median group** **(*n* = 2,612)**	**RC≥Median** **LDL-C < Median group** **(*n* = 2,684)**	**RC≥Median** **LDL-C≥Median group** **(*n* = 3,671)**	***p-*value**
**CLINICAL CHARACTERISTICS**						
Man(%)	8,658 (68.9%)	2,693 (74.9%)	1,777 (68.0%)	1,845 (68.7%)	2,343 (63.8%)	<0.001
Age (yrs)	61 (54,67)	62 (55,67)	61 (54,67)	61 (54,67)	60 (53,66)	<0.001
BMI (kg/m^2^)	25.86 (23.87,28.02)	25.55 (23.52,27.68)	25.47 (23.51,27.68)	26.19 (24.22,28.27)	26.09 (24.16,28.40)	<0.001
Hypertension, n (%)	7,906 (62.9%)	2,259 (62.8%)	1,500 (57.4%)	1,844 (68.7%)	2,303 (62.7%)	<0.001
Hypercholesterolemia, n (%)	9,894 (78.8%)	2,727 (75.8%)	2,113 (80.9%)	2,077 (77.4%)	2,977 (81.1%)	<0.001
Smoking, n (%)	5,801 (46.2%)	1,716 (47.7%)	1,202 (46.0%)	1,228 (45.8%)	1655 (45.1%)	0.143
Diabetes, n (%)	3,920 (31.2%)	1,178 (32.8%)	644 (24.7%)	1,000 (37.3%)	1,098 (29.9%)	<0.001
**LABORATORY PARAMETERS**						
SBP (mmHg)	130 (120,140)	128 (119,138)	130 (120,140)	129 (120,139)	130 (120,140)	<0.001
DBP (mmHg)	77 (70,84)	76 (70,82)	78 (70,85)	77 (70.84)	78 (70,85)	<0.001
FBG (mmol/L)	6.58 (5.39,13.61)	6.44 (5.35,12.36)	6.13 (5.28,10.42)	7.10 (5.52,16.91)	6.75 (5.47,16.42)	<0.001
HbA1C (%)	6.1 (5.6,6.9)	6.0 (5.6,6.8)	5.9 (5.6,6.6)	6.2 (5.7,7.2)	6.1 (5.7,7.1)	<0.001
TC (mmol/L)	3.97 (3.35,4.74)	3.18 (2.86,3.49)	4.42 (4.03,4.94)	3.59 (3.25,3.91)	4.91 (4.43,5.52)	<0.001
TG (mmol/L)	1.38 (0.99,2.00)	0.99 (0.78,1.25)	1.12 (0.88,1.41)	1.92 (1.43,2.65)	1.92 (1.47,2.56)	<0.001
LDL-C (mmol/L)	2.26 (1.76,2.91)	1.72 (1.44,1.97)	2.83 (2.50,3.31)	1.81 (1.55,2.04)	2.97 (2.58,3.51)	<0.001
HDL-C (mmol/L)	1.08 (0.93,1.27)	1.10 (0.94,1.28)	1.18 (1.02,1.38)	0.97 (0.84,1.15)	1.06 (0.93,1.24)	<0.001
RC (mmol/L)	0.51 (0.37,0.71)	0.37 (0.29,0.43)	0.37 (0.28,0.44)	0.67 (0.57,0.88)	0.73 (0.60,0.94)	<0.001
Non-HDL-C (mmol/L)	2.83 (2.26,3.58)	2.07 (1.78,2.33)	3.18 (2.85,3.67)	2.56 (2.27,2.81)	3.79 (3.36,4.39)	<0.001
Triglycerides >1.69 mmol/l+ HDL-C <1.03/1.29 mmol/l (in men/women)	11,214 (89.3%)	3,217 (89.5%)	2,348 (89.9%)	2,398 (89.3%)	3,251 (88.6%)	0.370
WBC ( × 10^∧^9/L)	6.72 (5.73,7.97)	6.58 (5.63,7.78)	6.73 (6.57,7.94)	6.75 (5.76,8.01)	6.83 (5.86,8.18)	<0.001
RBC ( × 10^∧^12/L)	4.63 (4.33,4.93)	4.59 (4.29,4.88)	4.65 (4.35,4.95)	4.60 (4.30,4.89)	4.68 (4.38,4.99)	<0.001
PLT ( × 10^∧^9/L)	221 (188,260)	213 (180,249)	227 (194,265)	214 (184,256)	231 (196,270)	<0.001
Hb (g/L)	143 (133,154)	142 (132,152)	145 (134,155)	142 (131,152)	145 (134,156)	<0.001
PT (S)	11.4 (10.9,11.9)	11.6 (11.1,12.1)	11.4 (11.0,11.9)	11.3 (10.8,11.8)	11.2 (10.8,11.7)	<0.001
ATPP (S)	32.5 (30.3,34.8)	32.6 (30.4,34.9)	32.6 (30.3,34.7)	32.3 (30.3,34.7)	32.4 (30.3,34.8)	0.157
BNP (pg/ml)	28 (15,58)	30 (16,59)	27 (15,57)	27 (14,55)	28 (14,61)	0.008
Hs-CRP (mg/L)	1.10 (0.52,2.74)	0.75 (0.39,1.87)	1.18 (0.56,3.02)	1.10 (0.54,2.60)	1.51 (0.72,3.53)	<0.001
Homocysteine (umol/L)	12.3 (9.9,15.3)	12.3 (10.0,15.3)	12.7 (10.4,15.7)	12.1 (9.7,15.0)	12.1 (9.8,15.4)	<0.001
Uric acid (umol/L)	333.3 (280.4,393.3)	322.5 (271.1,376.0)	322.8 (273.1,379.3)	342.9 (289.1,408.0)	346.3 (290.0,406.8)	<0.001
Creatinine (umol/L)	70.2 (60.9,80.5)	70.7 (62.1,80.0)	68.9 (59.7,79.2)	71.7 (61.8,82.1)	69.9 (60.1,80.5)	<0.001
**CLINICAL PRESENTATION, N (%)**						
Non-CAD	2,086 (16.6%)	485 (13.5%)	608 (23.3%)	386 (14.4%)	607 (16.5%)	<0.001
Stable CAD	241 (1.9%)	101 (2.8%)	47 (1.8%)	37 (1.4%)	56 (1.5%)	<0.001
ACS	10,236 (81.5%)	3,010 (83.7%)	1,957 (74.9%)	2,261 (84.2%)	3,008 (81.9%)	<0.001
Unstable angina	9,063 (72.1%)	2,783 (77.4%)	1,684 (64.5%)	2,061 (76.8%)	2,535 (69.1%)	<0.001
NSTEMI	666 (5.3%)	121 (3.4%)	156 (6.0%)	107 (4.0%)	282 (7.7%)	<0.001
STEMI	507 (4.0%)	106 (2.9%)	117 (4.5%)	93 (3.5%)	191 (5.2%)	<0.001

*Values are median or n (%)*.

*BMI, body mass index; SBP, systolic blood pressure; DBP, diastolic blood pressure; FBG, fasting blood glucose; HbA1C, glycosylated hemoglobin A1C; TC, total cholesterol; TG, triglyceride; LDL-C, low density lipoprotein cholesterol; HDL-C, high density lipoprotein cholesterol; WBC, white blood cell; RBC, red blood cell; PLT, platelets; PT, prothrombin time; ATPP, aqueous two-phase partitioning; BNP, brain natriuretic peptide; Hs-CRP, hyper-sensitive C-reactive protein; CAD, coronary artery disease; ACS, acute coronary syndrome; STEMI, ST-segment elevation myocardial infarction; NSTEMI, non-ST-segment elevation myocardial infarction*.

The baseline clinical characteristics of patients compared by the groups with the concordant and discordant values of RC vs. LDL-C is shown in Table 1. There were significant differences in age, gender, hypertension, smoking, diabetes, and other laboratory parameters across the four groups. Furthermore, the patients with a high RC index were more likely to have a higher FBG and WBC, while the patients with a high LDL-C index were more likely to have a higher hs-CRP. Similar patterns were also observed in the non-HDL-C vs. LDL-C and the RC vs. non-HDL-C groups, which are separately shown in Supplementary Tables 1, 2.

### The Correlation Analyses and Multivariable Logistic Regression Analyses (Before and After PSM)

As Spearman's correlation analyses before PSM showed (Table 2), the RC index was positively related to FBG, HbA1C, and hs-CRP (*r* = 0.098, 0.122, and 0.172, respectively, with all *p* < 0.001).

**Table 2:** Spearman's correlation coefficients of RC level with covariates.

**Variable**	**Correlation coefficient**	***p*** **-value**
Age	−0.071	<0.001
BMI	0.123	<0.001
FBG	0.098	<0.001
HbA1C	0.122	<0.001
LDL-C	0.193	<0.001
Non-HDL-C	0.488	<0.001
Hs-CRP	0.172	<0.001

*BMI, body mass index; FBG, fasting blood glucose; HbA1C, glycosylated hemoglobin A1C; LDL-C, low density lipoprotein cholesterol; HDL-C, high density lipoprotein cholesterol; Hs-CRP, hyper-sensitive C-reactive protein*.

**Figure 2:**
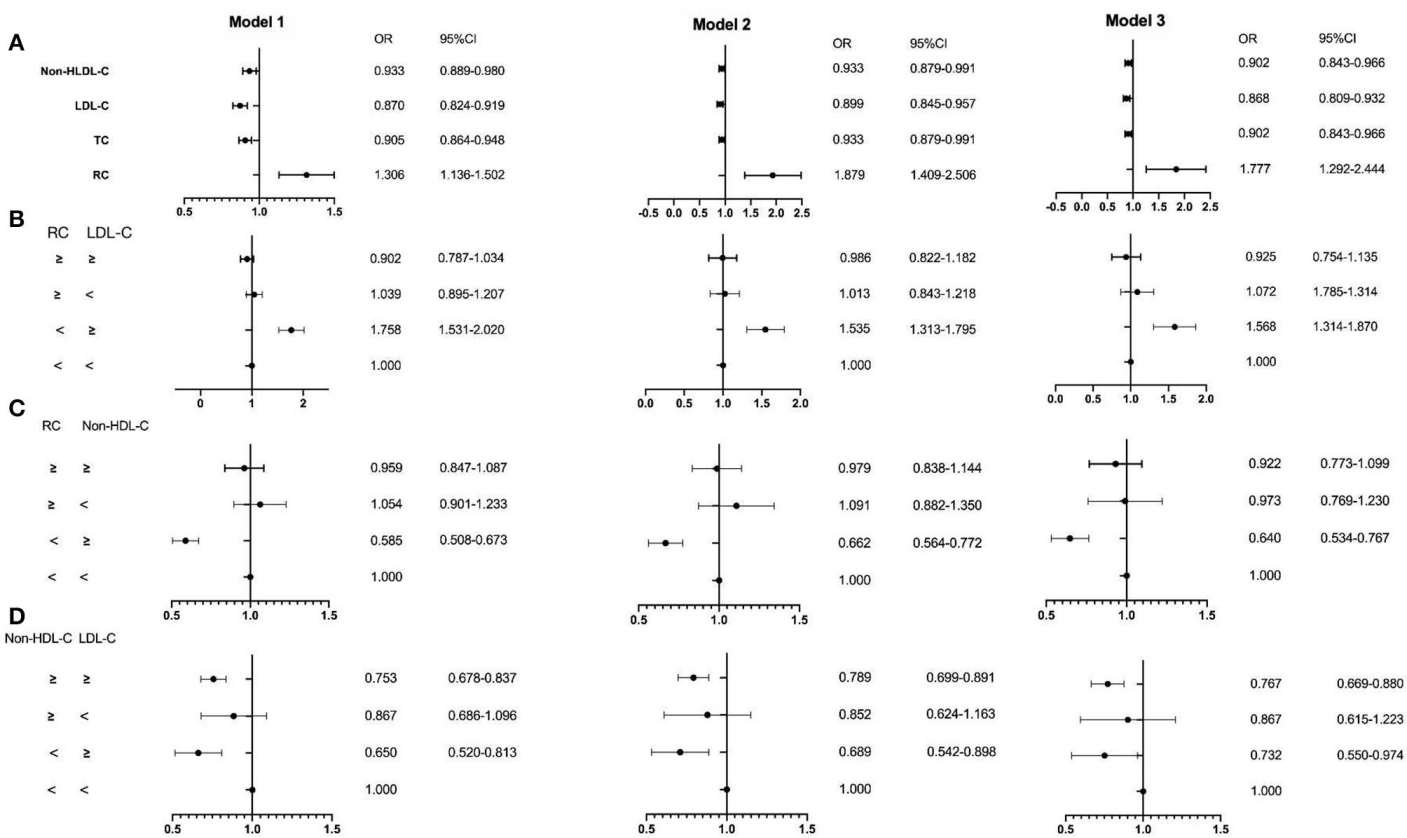
The results of the multivariable logistic regression analyses before propensity score matching (PSM). **(A)** Non-high-density lipoprotein cholesterol (non-HDL-C), low-density lipoprotein cholesterol (LDL-C), total cholesterol (TC), and remnant cholesterol RC; **(B)** the RC and LDL-C group; **(C)** the RC and non-HDL-C group; **(D)** the non-HDL-C and LDL-C group. Model 1: age, gender, hypertension, hypercholesterolemia, smoking, diabetes, and body mass index (BMI); Model 2: Model 1, systolic blood pressure (SBP), diastolic blood pressure (DBP), fasting blood glucose (FBG), glycosylated hemoglobin A1C (HbA1C), HDL-C, and triglyceride (TG); Model 3:Model 2, creatinine, uric acid, hypersensitive C-reactive protein (hs-CRP), brain natriuretic peptide (BNP), white blood cell (WBC), and homocysteine.

**Figure 3:**
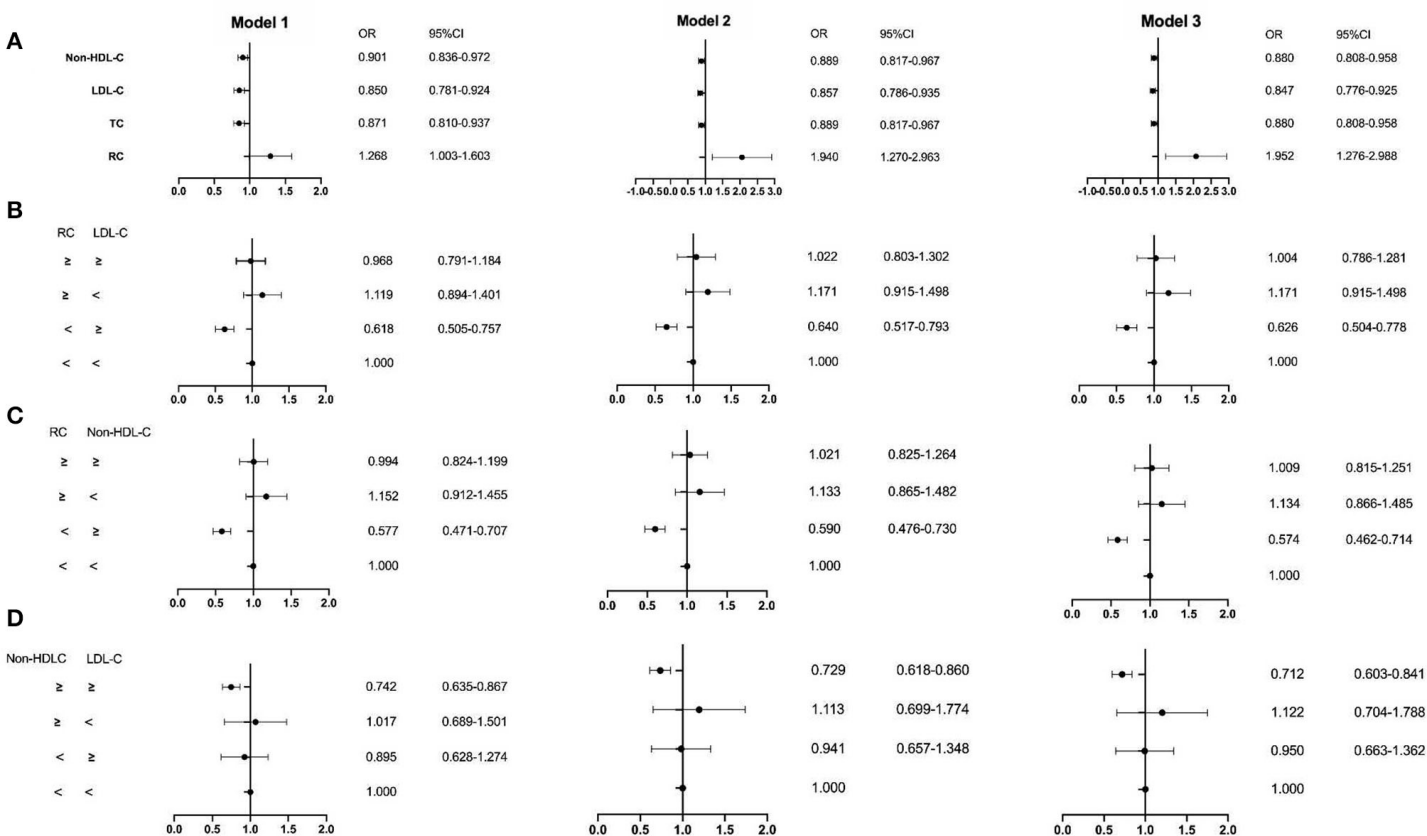
The result of the multivariable logistic regression analyses after PSM. **(A)** Non-HDL-C, LDL-C, TC, and RC; **(B)** the RC and LDL-C group; **(C)** the RC and non-HDL-C group; **(D)** the non-HDL-C and LDL-C group. Model 1: age, gender, hypertension, hypercholesterolemia, smoking, diabetes, and BMI; Model 2: Model 1, SBP, DBP, FBG, HbA1C, HDL-C, and TG; Model 3:Model 2, creatinine, uric acid, hs-CRP, BNP, WBC, and homocysteine.

The ROC curve showed the cut-off point of RC for PSM was 0.415 mmol/L. After PSM, there were 5,252 patients being enrolled, with 4,298 in the CAD group and 954 in the non-CAD group. The new median values of RC, LDL-C, and non-HDL-C were 0.42, 2.13, and 2.63 mmol/L. Then, the multivariate logistic regression analyses were performed to investigate the associations of independent confounders with CAD, before and after PSM. The models of analyses were as followed: model 1: age, gender, hypertension, hypercholesterolemia, smoking, diabetes, and BMI; model 2: model 1, SBP, DBP, FBG, HbA1C, HDL-C, and TG; model 3: model 2, CR, UA, hs-CRP, BNP, WBC, and homocysteine. After adjustment for the traditional predictors, the results illustrated that after PSM, the level of RC (*OR* = 1.952, *CI* = 1.276–2.988, *p* = 0.002) was an independent risk factor for CAD. While the *OR* of TC was 0.880 (*CI* = 0.808–0.958, *p* =0.003), the *OR* of LDL-C was 0.847 (*CI* = 0.776–0.925, *p* < 0.001), and the *OR* of non-HDL-D was 0.880 (*CI* = 0.808–0.958, *p* = 0.003) (Figures 2, 3 and Table 3). Furthermore, according to the concordance/discordance groups analyses, the *OR* of the low RC/high LDL-C group was 0.626 (*CI* = 0.504–0.778, *p* < 0.001) and the *OR* of low RC/high non-HDL-C group was 0.574 (*CI* = 0.462–0.714, *p* < 0.001), while the *OR* of high non-HDL-C/high LDL-C group was 0.712 (*CI* = 0.603–0.841, *p* < 0.001).

**Table 3:** The multivariate logistic regression analyses of the risk of coronary artery disease (CAD).

**Variable**	**OR (95% CI)**					
	**Before PSM**			**After PSM**		
	**Model 1**	**Model 2**	**Model 3**	**Model 1**	**Model 2**	**Model 3**
RC	1.306 (1.136–1.502) ^†^	1.879 (1.409–2.506) ^†^	1.777 (1.292–2.444) ^†^	1.268 (1.003–1.603) ^§^	1.940 (1.270–2.963) ^‡^	1.952 (1.276–2.988) ^‡^
TC	0.905 (0.864–0.948) ^†^	0.933 (0.879–0.991) ^§^	0.902 (0.843–0.966) ^‡^	0.871 (0.810–0.937) ^†^	0.889 (0.817–0.967) ^‡^	0.880 (0.808–0.958) ^‡^
LDL-C	0.870 (0.824–0.919) ^†^	0.899 (0.845–0.957) ^‡^	0.868 (0.809–0.932) ^†^	0.850 (0.781–0.924) ^†^	0.857 (0.786–0.935) ^†^	0.847 (0.776–0.925) ^†^
Non-HDL-C	0.933 (0.889–0.980) ^‡^	0.933 (0.879–0.991) ^§^	0.902 (0.843–0.966) ^‡^	0.901 (0.836–0.972) ^‡^	0.889 (0.817–0.967) ^‡^	0.880 (0.808–0.958) ^‡^
**RC/LDL**						
Low/low (referent)	-	-	-	-	-	-
Low/high	1.758 (1.531–2.020) ^†^	1.535 (1.313–1.795) ^†^	1.568 (1.314–1.870) ^†^	0.618 (0.505–0.757) ^†^	0.640 (0.517–0.793) ^†^	0.626 (0.504–0.778) ^†^
High/low	1.039 (0.895–1.207)	1.013 (0.843–1.218)	1.072 (0.875–1.314)	1.119 (0.894–1.401)	1.171 (0.915–1.498)	1.171 (0.915–1.498)
High/high	0.902 (0.787–1.034)	0.986 (0.822–1.182)	0.925 (0.754–1.135)	0.968 (0.791–1.184)	1.022 (0.803–1.302)	1.004 (0.786–1.281)
**RC/Non-HDLC**						
Low/low (referent)	-	-	-	-	-	-
Low/high	0.585 (0.508–0.673) ^†^	0.662 (0.564–0.776) ^†^	0.640 (0.534–0.767) ^†^	0.577 (0.471–0.707) ^†^	0.590 (0.476–0.730) ^†^	0.574 (0.462–0.714) ^†^
High/low	1.054 (0.901–1.233)	1.091 (0.882–1.350)	0.973 (0.769–1.230)	1.152 (0.912–1.455)	1.133 (0.865–1.482)	1.134 (0.866–1.485)
High/high	0.959 (0.847–1.087)	0.979 (0.838–1.144)	0.922 (0.773–1.099)	0.994 (0.824–1.199)	1.021 (0.825–1.264)	1.009 (0.815–1.251)
**Non-HDLC/LDL**						
Low/low (referent)	-	-	-	-	-	-
Low/high	0.650 (0.520–0.813) ^†^	0.698 (0.542–0.898) ^‡^	0.732 (0.550–0.974) ^§^	0.895 (0.628–1.274)	0.941 (0.657–1.348)	0.950 (0.663–1.362)
High/low	0.867 (0.686–1.096)	0.852 (0.624–1.163)	0.867 (0.615–1.223)	1.017 (0.689–1.501)	1.113 (0.699–1.774)	1.122 (0.704–1.788)
High/high	0.753 (0.678–0.837) ^†^	0.789 (0.699–0.891) ^†^	0.767 (0.669–0.880) ^†^	0.742 (0.635–0.867) ^†^	0.729 (0.618–0.860) ^†^	0.712 (0.603–0.841) ^†^

† *P < 0.001*.

‡ *P < 0.01*.

§ *P < 0.05*.

*Model 1: age+gender+hypertension+hypercholesterolemia+smoking+diabetes+body mass index (BMI)*.

*Model 2: Model 1+SBP+DBP+FBG+HbA1C+HDL+TG*.

*Model 3: Model 2+CR+UA+HsCRP+BNP+WBC+homocysteine*.

*TC, total cholesterol; TG, triglyceride; LDL-C, low density lipoprotein cholesterol; HDL-C, high density lipoprotein cholesterol; RC, remnant cholesterol*.

### The Subgroup Analyses (After PSM)

The subgroup analyses were performed to explore the *p-*value for interaction between the RC and other risk factors (Table 4). The results indicated that there was an interconnection between the RC and age, gender, hypertension, and diabetes as different risk factors for CAD (with all *p* for interaction < 0.001, respectively).

**Table 4:** The subgroup analyses for the relationship between RC and other risk factors after propensity score matching (PSM).

**Variables**	**RC**			
	**N**	**Odds Ratio (95%CI)**	***p*** **-value**	***p*****-value** **for interaction**
**Age**				
<75	4,959	1.190 (0.948–1.493)	0.133	<0.001
≥75	293	4.913 (0.795–30.360)	0.087	
**Sex**				
Men	3,798	1.213 (0.902–1.629)	0.201	<0.001
Women	1,454	1.259 (0.870–1.823)	0.222	
**Hypertension**				
No	2,080	1.135 (0.838–1.538)	0.413	<0.001
Yes	3,172	1.299 (0.953–1.806)	0.119	
**Diabetes**				
No	3,616	1.140 (0.882–1.472)	0.317	<0.001
Yes	1,636	1.364 (0.823–2.260)	0.228	

## Discussion

In this study, we elicited three major findings. First, the elevated level of RC was positively correlated with CAD. Furthermore, the multivariate logistic regression analyses indicated that RC was a risk factor for CAD, independently. Second, the level of RC was more capable to reflect the risk of CAD than LDL-C or non-HDL-C. Finally, there was an interaction relationship between RC and age, gender, hypertension, and diabetes in CAD progression.

### The Relationship Between RC and CAD

As far as we know, hypercholesterolemia is a common lipid disorder related to the increasing incidence of CAD (5). Because of the residual risk in the statin-treated patients with a seemingly optimal low level of LDL-C (18), more and more studies raised the hypothesis that there was a strong association between RC and CAD risk (8, 19, 20). The TRLs, also known as very-low-density lipoproteins (VLDL), intermediate-density lipoproteins (IDL), and their remnants, are always considered harmful for cardiovascular health (21, 22). Nordestgaard et al. demonstrated that it was cholesterol, but not triglycerides may cause atherosclerosis in the TRLs. Furthermore, the healthy participants with a high remnant-C level were more likely to have a greater risk of incident CAD, independently (23). *In-vitro* and animal studies indicated that the potential mechanism for this phenomenon may be that the remnant-C content could enter and get trapped in the arterial wall and have an influence as same as LDL-C (24–26). It could be taken up by the macrophages or smooth muscle cells (SMCs), then further induces inflammation, which plays an important role in the process of plaque initiation, progression, and rupture (27, 28). Moreover, in addition to LDL-C, non-HDL-C TRLs. Therefore, it is not surprising to find RC and non-HDL-C play an important role in the process of CAD.

Several cohort studies (11, 12, 29) and randomized clinical trials (10) investigating the relationship between RC, non-HDL-C, and the outcomes of CAD have been conducted over recent years. Castañer et al. (10) enrolled 6,901 patients from the Prevención con Dieta Mediterránea (PREDIMED) study, while Langsted et al. enrolled patients from the Copenhagen General Population Study (CGPS), a cohort study consisting of 109,574 individuals (29). Both the studies showed that the high level of remnant-C was associated with the MACEs. In addition to the CGPS, Varbo et al. included the patients from the CCHS Copenhagen City Heart Study (CCHS) and the Copenhagen Ischemic Heart Disease Study (CIHDS), a total of 73,513 subjects. It demonstrated that a 1 mmol/L (39 mg/dl) non-fasting remnant cholesterol increase may lead to a 2.8-fold causal risk for ischemic heart disease (12). Moreover, Johannesen et al. (11) suggested that non-HDL-C, but not LDL-C, was associated with an increased risk of all-cause mortality of CAD. Similarly, with the different models of multivariate logistic regression analyses, our study concluded that RC was an independent causal risk for CAD, which was consistent with the results of former studies.

### RC, LDL-C, and Non-HDL-C

Furthermore, the concordance/discordance groups analyses were adopted to compare the capacity of predicting the CAD risk between the RC and LDL-C or non-HDL-C, because of the tight correlation in those indexes and the hypothesis that RC could induct the elevated levels of other atherogenic lipoproteins (30). By comparing the disagreements between RC and LDL-C or non-HDL-C, these analyses could show us the final consequences of the RC index. After PSM, the results illustrated that the low RC groups had less possibility to suffer from CAD. Moreover, as one of the predictors, the level of RC was more capable to reflect the risk of CAD than LDL-C and non-HDL-C.

### RC and Other CAD Risk Factors

The previous studies confirmed the potential mechanism of RC could be that it inducted the migration of mononuclear cells and macrophage in the endothelial cells and promoted the occurrence of inflammation (31, 32). Spearman's correlation analyses of our study indicated that the level of RC in serum was positively related to hs-CRP. This phenomenon was consistent with the results of previous studies. Meanwhile, we performed the subgroup analyses to examine the association between RC and other risk factors in the incident and progression of CAD, such as age, gender, hypertension, and diabetes status. To our knowledge, hypertension and insulin resistance (IR) have a strong relationship with inflammation or dyslipidemia, both have been generally recognized that could promote CAD progression (33). As the results showed, there was an interaction between RC and other risk factors. But the exact physiological mechanisms that how they affect others influence in CAD progression remain unknown.

### The Analyses of Conflict Results in Our Study

Our study showed that non-HDL-C, TC, and LDL-C were the protective factors for CAD, and the disease incidence of the high non-HDL-C/high LDL-C group was less than the low non-HDL-C/low LDL-C group, which were inconsistent with the clinical phenomenon and the results of existing research. All the conflict results in our study mentioned above may be attributed to: 1) our study was a retrospective study, while a cohort study or a randomized clinical trial could get much closer to the real-world scenarios and draw conclusions in line with the clinical phenomenon. 2) Most participation in this study were unstable patients with angina (72.1%), which may cause a deviation from the average value of all populations. 3) The study did not take the use of lipid-lowering drugs into consideration. It may be the potential reason for the conflict reasons.

## Limitations

Our present study has several limitations. 1) This study was a single-center, retrospective study. The study was also conducted with a nonrandomized sample, which probably restricted the generalizability of our results. Thus, there must be more multicenter randomized-controlled trials done to explore the relationship between RC and CAD in the future. 2) Our study only enrolled a Chinese population at a single hospital. 3) Our data were collected from the clinical database and directly measured RC has not become a routine test item in the clinical blood lipid testing. So, we can only get the level of calculated RC. 4) The study did not take the use of lipid-lowering drugs or socioeconomic status into consideration. 5) Finally, the detailed interaction relationship between RC and age, gender, hypertension, and diabetes needs further exploration.

## Conclusion

Hypercholesterolemia is a common lipid disorder related to the increasing incidence of CAD. Besides LDL-C, our study illustrated a significant association between RC and CAD. Furthermore, the level of RC was more capable to reflect the risk of CAD than LDL-C or non-HDL-C. Moreover, there was an interaction relationship between the RC and age, gender, hypertension, and diabetes in CAD progression. But whether non-HDL-C could be an independent risk factor for CAD was not reflected in this study. And the exact interaction between the RC and other CAD risk factors remains unclear. Further studies of RC and its exact mechanisms in the incident and progression of CAD need to be conducted to promote the development of lipid-lowering therapy for patients with CAD.

## Data Availability Statement

The original contributions presented in the study are included in the article/Supplementary Material, further inquiries can be directed to the corresponding author.

## Ethics Statement

The studies involving human participants were reviewed and approved by Beijing Anzhen Hospital Ethics Committee of Capital Medical University. The patients/participants provided their written informed consent to participate in this study.

## Author Contributions

WK carried out the experiments, acquired the data, and wrote the first draft of the manuscript. DY carried out the experiments and wrote sections of the manuscript. GW, WR, YJ, LX, and SH recruited the subjects, performed the assessments of patients, and critically reviewed the manuscript for intellectual content. WK, DY, WR, and YJ performed the statistical analyses. GH conceived and designed the study and handled funding and supervision. All the authors read and approved the final manuscript.

## Funding

This work was supported by the China National Natural Scientific Foundation (grant nos. 81973841 and 81573744). Efficacy and safety of transcatheter aortic valve replacement vs. surgical aortic valve replacement in elderly patients with severe aortic stenosis (the Beijing Municipal Health Commission: jing 19-15). The Capital's Funds for Health Improvement and Research (CFH 2020-2-2063) KM200910025012 and the Beijing Municipal Natural Science Foundation (grant no. 7202041).

## Conflict of Interest

The authors declare that the research was conducted in the absence of any commercial or financial relationships that could be construed as a potential conflict of interest.

## Publisher's Note

All claims expressed in this article are solely those of the authors and do not necessarily represent those of their affiliated organizations, or those of the publisher, the editors and the reviewers. Any product that may be evaluated in this article, or claim that may be made by its manufacturer, is not guaranteed or endorsed by the publisher.

## Abbreviations

CAD: coronary artery disease
RC: remnant cholesterol
BMI: body mass index
SBP: systolic blood pressure
DBP: diastolic blood pressure
FBG: fasting blood glucose
HbA1C: glycosylated hemoglobin A1C
TC: total cholesterol
TG: triglyceride
LDL-C: low density lipoprotein cholesterol
HDL-C: high density lipoprotein cholesterol
WBC: white blood cell
RBC: red blood cell
PLT: platelets
PT: prothrombin time
ATPP: aqueous two-phase partitioning
BNP: brain natriuretic peptide
hs-CRP: hypersensitive C-reactive protein
ACS: acute coronary syndrome
STEMI: ST-segment elevation myocardial infarction
NSTEMI: non-ST-segment elevation myocardial infarction
ROC: receiver-operating characteristic curve.
