# Supplementary material for: Association of Increased Remnant Cholesterol and the Risk of Coronary Artery Disease: A Retrospective Study
Source: Front Cardiovasc Med. 2021 Oct 29;8:740596. doi: 10.3389/fcvm.2021.740596 (PMC8585757; doi:10.3389/fcvm.2021.740596)
Supplement: Supplementary file 1 [file Table_1.DOCX]

Supplemental Table 1 Baseline clinical characteristics and laboratory parameters of patients according to Non-HDL-C and LDL-C index categories

| Variables | Non-HDL-C＜Median  LDL-C< Median group  (n=5642) | Non-HDL-C＜Median  LDL-C≥ Median group  (n=601) | Non-HDL-C≥Median  LDL-C＜ Median group  (n=638) | Non-HDL-C≥Median  LDL-C≥ Median group  (n=5682) | *p*  value |
| --- | --- | --- | --- | --- | --- |
| Clinical Characteristics |  |  |  |  |  |
| Man(%) | 4096(72.6%) | 419(69.7%) | 442(69.3%) | 3701(65.3%) | ＜0.001 |
| Age (yrs) | 62(55,67) | 61(55,68) | 59(52,66) | 60(53,66) | ＜0.001 |
| BMI (kg/m^2^) | 25.77(23.78,27.77) | 25.14(23.14,27.54) | 26.46(24.44,28.71) | 25.91(23.99,28.22) | ＜0.001 |
| Hypertension, n (%) | 3665(65.0%) | 375(62.4%) | 438(68.7%) | 3428(60.3%) | ＜0.001 |
| Hypercholesterolemia,n (%) | 4278(75.8%) | 492(81.9%) | 526(82.4%) | 4598(80.9%) | ＜0.001 |
| Smoking ,n (%) | 2626(46.5%) | 286(47.6%) | 318(49.8%) | 2571(45.2%) | 0.102 |
| Diabetes,n (%) | 1940(34.4%) | 166(27.6%) | 238(37.3%) | 1576(27.7%) | ＜0.001 |
| Laboratory parameters |  |  |  |  |  |
| SBP (mmHg) | 128(119,138) | 130(120,140) | 130(120,140) | 130(120,140) | ＜0.001 |
| DBP (mmHg) | 76(70,83) | 77(70,85) | 80(71,87) | 78(70,85) | ＜0.001 |
| FBG (mmol/L) | 6.33(5.40,13.61) | 6.23(5.24,11.61) | 7.37(5.61,17.55) | 6.50(5.39,13.42) | ＜0.001 |
| HbA1C (%) | 6.1(5.7,6.9) | 6.0(5.5,6.7) | 6.3(5.7,7.5) | 6.0(5.6,6.9) | ＜0.001 |
| TC (mmol/L） | 3.28(2.94,3.58) | 3.86(3.70,4.08) | 4.09(3.89,4.40) | 4.80(4.36,5.39) | ＜0.001 |
| TG (mmol/L） | 1.18(0.87,1.62) | 0.99(0.80,1.26) | 3.04(2.26,4.43) | 1.59(1.17,2.21) | ＜0.001 |
| LDL-C(mmol/L） | 1.73(1.46,1.97) | 2.35(2.30,2.44) | 2.06(1.87,2.17) | 2.99(2.64,3.50) | ＜0.001 |
| HDL-C(mmol/L） | 1.06(0.90,1.25) | 1.17(1.00,1.37) | 0.93(0.81,1.08) | 1.10(0.95,1.30) | ＜0.001 |
| Remnant-C(mmol/L) | 0.44(0.34,0.56) | 0.32(0.21,0.40) | 1.08(0.83,1.55) | 0.59(0.44,0.81) | ＜0.001 |
| Non-HDL-C(mmol/L) | 2.20(1.89,2.46) | 2.71(2.63,2.78) | 3.08(2.93,3.36) | 3.63(3.22,4.21) | ＜0.001 |
| Triglycerides >1.69 mmol/l＋ HDL-C <1.03/1.29 mmol/l (in men/women) | 5051(89.5%) | 543(90.3%) | 564(88.4%) | 5056(89.0%) | 0.551 |
| WBC (×10^9/L) | 6.63(5.66,7.86) | 6.67(5.74,7.95) | 6.82(5.82,8.12) | 6.80(5.77,8.11) | ＜0.001 |
| RBC (×10^12/L) | 4.58(4.29,4.88) | 4.61(4.33,4.91) | 4.66(3.35,4.94) | 4.67(4.38,4.98) | ＜0.001 |
| PLT (×10^9/L) | 213(181,251) | 222(193,261) | 219(186,252) | 230(196,296) | ＜0.001 |
| Hb(g/L) | 142(132,152) | 144(133,154) | 143(133,154) | 145(134,156) | ＜0.001 |
| PT(Sec) | 11.5(11.0,12.0) | 11.5(11.0,12.0) | 11.1(10.6,11.6) | 11.3(10.8,11.8) | ＜0.001 |
| ATPP(Sec) | 32.5(30.4,34.8) | 32.6(30.3,34.7) | 32.4(30.1,34.5) | 32.4(30.3,34.8) | 0.475 |
| BNP(pg/ml) | 29(16,58) | 29(15,55) | 25(13,53) | 27(15,60) | 0.009 |
| Hs-CRP(mg/L) | 0.85(0.43,2.05) | 1.01(0.50,2.51) | 1.34(0.70,3.24) | 1.43(0.65,3.42) | ＜0.001 |
| Homocysteine (umol/L) | 12.2(9.9,15.2) | 12.9(10,7,15.5) | 12.2(10.8,15.1) | 12.3(9.9,15.5) | 0.006 |
| Uric acid(umol/L) | 328.0(275.3,386.3) | 319.1(270.6,369.3) | 355.1(300.3,419.5) | 338.0(284.0,398.6) | ＜0.001 |
| Creatinine (umol/L) | 71.0(61.9,80.8) | 68.5(59.5,79.4) | 71.6(62.4,82.4) | 69.7(60.0,80.0) | ＜0.001 |
| Clinical presentation,n (%) |  |  |  |  |  |
| Non-CAD group | 770(13.6%) | 118(19.6%) | 101(15.8%) | 1097(19.3%) | ＜0.001 |
| Stable CAD | 136(2.4%) | 11(1.8%) | 2(0.3%) | 92(1.6%) | ＜0.001 |
| ACS | 4736(83.9%) | 472(78.5%) | 535(83.9%) | 4493(79.1%) | ＜0.001 |
| Unstable angina | 4378(77.6%) | 421(70.0%) | 466(73.0%) | 3798(66.8%) | ＜0.001 |
| NSTEMI | 189(3.3%) | 24(4.0%) | 39(6.1%) | 414(7.3%) | ＜0.001 |
| STEMI | 169(3.0%) | 27(4.5%) | 30(4.7%) | 281(4.9%) | ＜0.001 |

^a.^ Values are median or n (%).

^b.^ Abbreviations :BMI, body mass index; SBP, Systolic blood pressure; DBP, Diastolic blood pressure; FBG , Fasting blood glucose; HbA1C, Glycosylated hemoglobin A1C; TC, total cholesterol; TG, triglyceride; LDL-C, Low density lipoprotein cholesterol; HDL-C, High density lipoprotein cholesterol; WBC, white blood cell; RBC, red blood cell; PLT, Platelets; PT, Prothrombin time; ATPP, Aqueous two-phase partitioning; BNP, Brain Natriuretic Peptide; Hs-CRP, hyper-sensitive C-reactive protein; CAD, Coronary artery disease; ACS, Acute Coronary Syndrome; STEMI, ST-segment elevation myocardial infarction; NSTEMI, non-ST-segment elevation myocardial infarction
