# Supplementary material for: Association of Increased Remnant Cholesterol and the Risk of Coronary Artery Disease: A Retrospective Study
Source: Front Cardiovasc Med. 2021 Oct 29;8:740596. doi: 10.3389/fcvm.2021.740596 (PMC8585757; doi:10.3389/fcvm.2021.740596)
Supplement: Supplementary file 2 [file Table_2.DOCX]

Supplemental Table 2 Baseline clinical characteristics and laboratory parameters of patients according to RC and Non-HDL-C index categories

| Variables | RC＜Median  Non-HDL-C< Median group  （n=4177） | RC＜Median  Non-HDL-C≥ Median group  （n=2031） | RC≥Median  Non-HDL-C＜ Median group  （n=2066） | RC≥Median  Non-HDL-C≥ Median group  （n=4289） | *p*  value |
| --- | --- | --- | --- | --- | --- |
| Clinical Characteristics |  |  |  |  |  |
| Man(%) | 3101(74.2%) | 1369(67.4%) | 1414(68.4%) | 2774(64.7%) | ＜0.001 |
| Age (yrs) | 62(55,67) | 61(54,67) | 61(55,67) | 60(53,66) | ＜0.001 |
| BMI (kg/m^2^) | 25.51(23.45,27.68) | 25.59(23.59,27.73) | 26.06(24.16,28.09) | 26.17(24.21,28.40) | ＜0.001 |
| Hypertension, n (%) | 2616(62.6%) | 1143(56.3%) | 1424(68.9%) | 2723(63.5%) | ＜0.001 |
| Hypercholesterolemia,n (%) | 3205(76.7%) | 1635(80.5%) | 1565(75.8%) | 3489(81.3%) | ＜0.001 |
| Smoking ,n (%) | 1995(47.8%) | 923(45.4%) | 917(44.4%) | 1966(45.8%) | 0.057 |
| Diabetes,n (%) | 1338(32.0%) | 484(23.8%) | 768(37.2%) | 1330(31.0%) | ＜0.001 |
| Laboratory parameters |  |  |  |  |  |
| SBP (mmHg) | 129(119,138) | 130(120,140) | 128(119,138) | 130(120,140) | ＜0.001 |
| DBP (mmHg) | 76(70.82) | 78(70,85) | 77(70,84) | 78(70,85) | ＜0.001 |
| FBG (mmol/L) | 6.40(5.33,12.17) | 6.14(5.29,10.25) | 7.01(5.50,17.28) | 6.83(5.49,16.34) | ＜0.001 |
| HbA1C (%) | 6.0(5.6,6.8) | 5.9(5.6,6.5) | 6.2(5.7,7.1) | 6.2(5.7,7.1) | ＜0.001 |
| TC (mmol/L） | 3.28(2.91,3.64) | 4.61(4.24,5.08) | 3.46(3.14,3.70) | 4.79(4.30,5.44) | ＜0.001 |
| TG (mmol/L） | 0.98(0.78,1.24) | 1.16(0.91,1.45) | 1.73(1.33,2.25) | 2.05(1.54,2.81) | ＜0.001 |
| LDL-C(mmol/L） | 1.80(1.49,2.10) | 3.01(2.71,3.47) | 1.74(1.49,1.97) | 2.83(2.42,3.39) | ＜0.001 |
| HDL-C(mmol/L） | 1.11(0.95,1.29) | 1.19(1.02,1.38) | 0.99(0.85,1.18) | 1.04(0.91,1.22) | ＜0.001 |
| Remnant-C(mmol/L) | 0.36(0.28,0.43) | 0.38(0.30,0.45) | 0.61(0.55,0.73) | 0.77(0.62,1.01) | ＜0.001 |
| Non-HDL-C(mmol/L) | 2.15(1.84,2.46) | 3.37(3.06,3.82) | 2.43(2.18,2.64) | 3.68(3.24,4.31) | ＜0.001 |
| Triglycerides >1.69 mmol/l＋ HDL-C <1.03/1.29 mmol/l (in men/women) | 3742(89.6%) | 1823(89.8%) | 1852(89.6%) | 3797(88.5%) | 0.296 |
| WBC (×10^9/L) | 6.59(5.64,7.79) | 6.74(5.64,7.95) | 6.72(5.73,7.99) | 6.83(5.85,8.17) | ＜0.001 |
| RBC (×10^12/L) | 4.60(4.30,4.88) | 4.66(4.36,4.97) | 4.58(4.28,4.88) | 4.68(4.38,4.98) | ＜0.001 |
| PLT (×10^9/L) | 214(181,251) | 228(195,266) | 213(183,258) | 229(195,267) | ＜0.001 |
| Hb(g/L) | 143(132,152) | 145(134,155) | 141(131,152) | 144(134,155) | ＜0.001 |
| PT(Sec) | 11.6(11.1,12.1) | 11.4(10.9,11.9) | 11.3(10.9,11.8) | 11.2(10.8,11.7) | ＜0.001 |
| ATPP(Sec) | 32.6(30.4,34.9) | 32.6(30.3,34.8) | 32.3(30.3,34.7) | 32.4(30.2,34.7) | 0.225 |
| BNP(pg/ml) | 30(16,59) | 27(15,59) | 28(15,55) | 27(14,59) | 0.023 |
| Hs-CRP(mg/L) | 0.79(0.40,1.97) | 1.25(0.56,3.20) | 1.05(0.51,2.30) | 1.50(0.72,3.50) | ＜0.001 |
| Homocysteine (umol/L) | 12.4(10.1,15.3) | 12.6(10.4,15.7) | 12.0(9.7,15.0) | 12.1(9.8,15.3) |  |
| Uric acid(umol/L) | 321.8(270.7,375.0) | 324.2(274.2,383.0) | 339.7(285.8,404.3) | 347.5(291.4,408.1) | ＜0.001 |
| Creatinine (umol/L) | 70.4(61.7,79.9) | 69.1(60.0,79.2) | 71.7(61.6,82.0) | 70.0(60.3,80.8) | ＜0.001 |
| Clinical presentation,n (%) |  |  |  |  |  |
| Non-CAD group | 598(14.3%) | 495(24.4%) | 290(14.0%) | 703(16.4%) | ＜0.001 |
| Stable CAD | 110(2.6%) | 38(1.9%) | 37(1.8%) | 56(1.3%) | ＜0.001 |
| ACS | 3469(83.1%) | 1498(73.8%) | 1739(84.2%) | 3530(82.3%) | ＜0.001 |
| Unstable angina | 3195(76.5%) | 1272(62.6%) | 1604(77.6%) | 2992(69.8%) | ＜0.001 |
| NSTEMI | 143(3.4%) | 134(6.6%) | 70(3.4%) | 319(7.4%) | ＜0.001 |
| STEMI | 131(3.1%) | 92(4.5%) | 65(3.1%) | 219(5.1%) | ＜0.001 |

^a.^ Values are median or n (%).

^b.^ Abbreviations: BMI, body mass index; SBP, Systolic blood pressure; DBP, Diastolic blood pressure; FBG , Fasting blood glucose; HbA1C, Glycosylated hemoglobin A1C; TC, total cholesterol; TG, triglyceride; LDL-C, Low density lipoprotein cholesterol; HDL-C, High density lipoprotein cholesterol; WBC, white blood cell; RBC, red blood cell; PLT, Platelets; PT, Prothrombin time; ATPP, Aqueous two-phase partitioning; BNP, Brain Natriuretic Peptide; Hs-CRP, hyper-sensitive C-reactive protein; CAD, Coronary artery disease; ACS, Acute Coronary Syndrome; STEMI, ST-segment elevation myocardial infarction; NSTEMI, non-ST-segment elevation myocardial infarction
